# Development of a self-assessment tool to address the functioning of community-dwelling older adults in general practice: a validation study of the EFA23 questionnaire

**DOI:** 10.1186/s12875-024-02539-6

**Published:** 2024-08-02

**Authors:** Laura Rink, Caroline Floto, Katharina Apel, Maren Weiss, Elmar Stegmeier, Thomas Kühlein, Maria Sebastião

**Affiliations:** 1 Institute of General Practice, Friedrich-Alexander-Universität Erlangen-Nürnberg (FAU), Universitätsstr. 29, 91054 Erlangen, Germany; 2 Department of Psychology, SRH Wilhelm Löhe Hochschule Fürth, Fürth, Germany; 3 Koordinierungsgesellschaft Gesundheit, Aschau im Chiemgau, Germany

**Keywords:** International classification of functioning, Multimorbidity, Older adults, General practice, Functional health

## Abstract

**Background:**

Multimorbidity is increasingly prevalent among ageing patients, leading to reduced daily functioning. To address the challenges posed by multimorbidity in older adults, a person- and context-centred approach is needed. This study aimed to develop a questionnaire as a self-assessment tool for older adults focusing on functioning in general practice based on the International Classification of Functioning, Disability and Health (ICF).

**Methods:**

A mixed-methods approach was employed in the development and validation of the German EFA23 (*Erfassung Funktionaler Gesundheit im Alter – 23 Fragen; Assessing Functional Health in Old Age – 23 questions)* questionnaire. Based on an ICF subset developed in a preparatory phase and consensus study, questionnaire items were formulated and tested in a qualitative pretest, followed by a validation study. A workshop with general practitioners (GPs) was held to develop a supplementary manual for GPs on how to interpret the questionnaire results and discuss them with the patients.

**Results:**

A total of 69 items were developed and tested in the qualitative pretest with 15 respondents, resulting in 37 items for the validation study. The validation study, involving 237 older adults, showed the presence of one significant principal component. It demonstrated good internal consistency (Cronbach’s alpha = 0.967) and construct validity, showing positive correlations with established assessment tools. Descriptive statistics showed differences between the means of self-assessment by patients and an external GP assessment. The final EFA23 questionnaire consists of 23 items assessing limitations in functioning.

**Conclusions:**

The EFA23 questionnaire can be used as a valid self-assessment instrument to measure functioning in older adults, supporting a person- and context-centred approach in general practice.

**Supplementary Information:**

The online version contains supplementary material available at 10.1186/s12875-024-02539-6.

## Background

Multimorbidity can be seen as the norm among ageing patients [1]. With the presence of multiple chronic conditions being increasingly prevalent [2], patients often experience reduced levels of daily functioning [3, 4], meaning the extent to which people are able to perform actions and tasks in their living environment [5]. They face barriers in engaging in activities and managing the impact of their illnesses on various aspects of functioning [6, 7]. This highlights the limitations of a disease-focused approach when dealing with older multimorbid patients. Moreover, relying on isolated disease-specific guidelines can lead to overtreatment and adverse drug events, increasing the risks of falls, hospitalization, and even mortality [8–10]. To address the unique challenges posed by multimorbidity in older adults, a person- and context-centred approach is needed. This involves considering not only disease-related information but also information about functioning in terms of activities and participation, as well as treatment relevant for these patients [11–13]. A holistic approach, considering the broader context of patients’ lives, might provide a better guide for deciding on appropriate medical interventions than relying solely on disease-related information.

To place the functioning of older adults at the centre of care, it must first be adequately assessed. The International Classification of Functioning, Disability and Health (ICF) would be a suitable tool to describe functioning [14]. However, its mere size, with over 1,400 categories to describe a person, makes it impractical for primary healthcare settings. Therefore, we developed an ICF-based subset following a methodological approach of the ICF research branch [15] to reduce the ICF to a meaningful subset assessing the functioning of community-dwelling older adults aged 75 and above in general practice. This approach encompasses the integration of four preparatory studies, identifying potentially relevant ICF categories in old age from four different perspectives: research [16], patients’ [17], experts’ [18], and clinical perspective [19]. Subsequently, the results of all studies were discussed in a consensus process by seven international experts, resulting in an ICF-based subset for older adults focusing on the ICF component activities and participation to address functioning in general practice [20].

The ICF serves as a classification system to describe functioning rather than a measuring tool [14]. A questionnaire can be employed to collect and assess standardized data on the effects of ageing on everyday life and functioning, focusing on individual areas of the patients according to their problems in certain fields. Self-assessment questionnaires can encourage patients to reflect on their health and address their thoughts and possible concerns in their own words [21, 22]. In this way, general practitioners (GPs) gain insights into functional areas that are particularly challenging for their older patients. These can be discussed with the patients to enable a more person-centred conversation [22], focusing on functional health.

The aim of this study was to translate the ICF-based subset into a manageable self-assessment tool for patients in Germany – the EFA23 (*Erfassung Funktionaler Gesundheit im Alter – 23 Fragen; Assessing Functional Health in Old Age – 23 questions)* questionnaire – that can be used even under tightly scheduled conditions in general practice to support communication processes in consultations.

## Methods

**Fig. 1:**
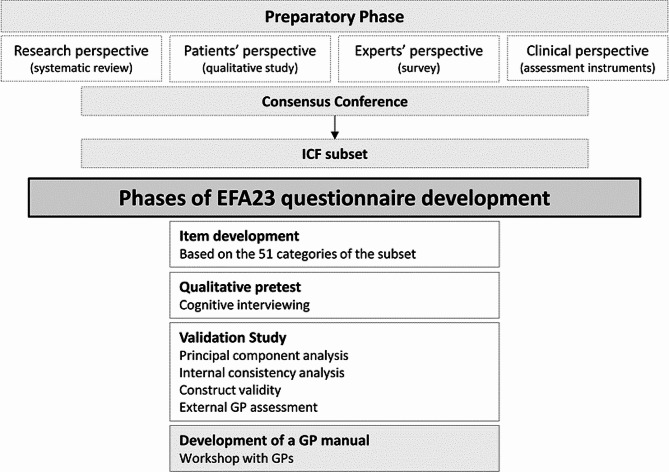
Development and validation steps of the EFA23 questionnaire

The theoretical construct of the EFA23 questionnaire for older adults aged 75 and above is given by the ICF framework [14]. Figure 1 shows the development and validation process of the questionnaire, following a mixed-methods approach.

### Preparatory phase: content validity

The four preparatory studies [16–19] and the involvement of various disciplines in the consensus study [20] provided different perspectives, forming the foundation for the selection of categories and the subsequent development of questionnaire items. The preparatory studies included patients’ [17] and GPs’ [18] perspectives, while in the consensus study experts from the disciplines of general practice, geriatrics, gerontology, physiotherapy, rehabilitation medicine, nursing sciences, and public health were involved [20]. Based on this, it can be assumed that the EFA23 questionnaire will comprehensively cover the construct to be measured in the self-assessment in its most important aspects.

### Item development

Since the ICF is written in a specific scientific language, it was necessary for the questionnaire to rephrase certain categories into lay language. A rough version of the questionnaire was designed and developed by the research team based on the results of the preparatory phase [16–19] and the consensus process [20]. The ICF differentiates in its categories between capacity (what a person can do) and performance (what is actually carried out). When formulating the items, we decided to ask about the capacity to capture the functional health of persons, while the performance would additionally depend on contextual factors.

For all items, we generated a three-point scale, with the values “yes”, “partly” and “no”, to measure the capacity. To determine whether a possible limitation in capacity has relevance for older adults and to identify individual problem areas more quickly in the consultation, an additional item was developed to capture whether the limitation in capacity is perceived as a problem. If “partly” or “no” is selected in the capacity item, this additional item should be rated dichotomously (“yes” or “no”).

For certain categories, we decided that employing a more detailed levelled question approach in the items would be more appropriate and would enhance patients’ understanding, e.g., for the ICF category *“d740 Formal relationships”*, we formulated the more detailed item *“I am able to contact service providers*,* such as a hairdresser or doctor”*. Moreover, for some categories, we formulated more than one item option to test in the next step. The draft version of the questionnaire contained a pool of possible items, at least one per category included in the ICF-based subset.

### Qualitative pretest

The preliminary version of the questionnaire was presented to respondents for a cognitive pretest using a qualitative design consisting of focus groups and one-on-one interviews. Following a semistructured interview approach, the pretest aimed to determine how the respondents interpret the items and how they come to a decision. Through the qualitative pretest, we used the “thinking aloud method” [23] to gather information about comprehensibility, readability, answer formation, and time required to complete the questionnaire. In addition, the suitability of the entire questionnaire, such as the layout and the order of the individual items, was discussed.

The interviews and focus groups took place between November and December 2021. Respondents were recruited from the general population through snowball sampling. Flyers were displayed in various neighbourhoods in the area in and around Erlangen with different socio-demographic structures, as well as in pharmacies and supermarkets. Due to the COVID-19 pandemic situation at the time in which the pretest was planned, the interviews had to be set up as online-only interviews. We aimed to interview approximately 15 persons in the pretest. Participants were offered a financial incentive of 50 €.

The main aim of the pretest was to check whether the items were formulated in a generally understandable way in the one-on-one interviews and additionally to weigh up several alternatives in the focus groups. As we expected difficulties for the older people in the target group aged 75 and above to participate in online cognitive interviews discussing various items, we decided to split the pretest into two phases. In the first phase, we discussed the questionnaire with younger participants to make a first selection and adjustment of items. In the second phase, the questionnaire was presented to older participants. We intentionally narrowed our focus to the critical questionnaire items from pretest phase one in phase two, recognizing that fewer items would alleviate stress, particularly for the more vulnerable group of respondents. In either phase one or phase two, both one-on-one interviews and a focus group were scheduled.

The participants in the second phase of the pretest did not necessarily have to be in the target group aged 75 and above, as we expected technical difficulties due to online participation for older respondents. We assumed that nevertheless the perspective of these older participants was adequately considered in the preparatory study [17]. In the subsequent validation, only people aged 75 and above were included.

All participants in the pretest had to be at least 18 years and fluent in German. All of them were informed about the aim of the study and gave written informed consent, followed by a thorough explanation about the aims and conditions of the study provided by the moderators before each interview and focus group.

All interviews and focus groups were conducted by two moderators (LR and MS, both female, research assistants). Both researchers are experienced in qualitative interview studies and have had specific professional training. The one-on-one interviews lasted between 30 and 60 min, and the focus groups lasted between 60 and 90 min. All interviews and focus groups were audio recorded. All the participants’ suggestions to rephrase or discard questionnaire items were transcribed in a short protocol, and the questionnaire was successively revised.

### Validation study

Participants for the validation study were recruited over ten months from May 2022 to February 2023. Based on the consensus study and the experts’ recommendation that the final questionnaire should have a maximum of 15–20 items, we aimed to recruit about 200 participants, following a methodologically recommended subject-to-item ratio of 10:1 [24]. Participants were offered a financial incentive of 30 €. Two general practices assisted in recruiting community-dwelling adults aged 75 years and above. Additional respondents were recruited through snowball sampling and flyers that were distributed inviting potential participants to contact the research team in case of interest in participating. After contacting the research team by phone or email, participants received the survey and a written consent form to complete and return. The survey encompassed sociodemographic and disease-related information, along with the EFA23 questionnaire and supplementary, already validated assessment tools utilized to assess the validity of the EFA23.

We chose the additional assessment tools to test the construct validity of the EFA23 questionnaire based on their (partly) similar concepts for the assessment of functional health: the *Instrumental activities of daily living scale* (IADL) for measuring functional independence in everyday tasks [25]; the *World Health Organization Disability Assessment Schedule* (WHODAS 2.0) for measuring disability and functioning [26]; the *Questionnaire to assess the areas Mobility*,* Self-Care and Domestic life* (MOSES) [27]; the *Standardized assessment of elderly people in primary care* (STEP) for addressing common health issues among independently living older adults in primary care [28]; and the *Functional Assessment Measure* (FAM) measuring the overall functional abilities in daily living [29].

#### Preparation of data

In the first step, we generated a new item for each original EFA23 questionnaire item to measure the degree of limitations in functioning. To achieve this, the capacity item was recoded, assigning values of 0 (“yes”), 1 (“partly”), and 2 (“no”). The second item, to evaluate whether the limitation in a capacity is a problem, was coded as 1 (problem = “yes”) and − 1 (problem = “no”). The newly created item was then computed by summing up the corresponding values. The resulting item, measuring the particular limitations in functioning, exhibited values from 0, indicating “no limitation”, to 3, representing “complete limitation” in functioning in the respective area.

#### Statistical analysis

Descriptive statistics, including frequency, mean, and standard deviation, were calculated for each of these newly generated items. Items that did not utilize the full range of response categories (from 0 = “no limitation” to 3 = “complete limitation”) were removed from further analysis to ensure the quality of data [30]. Subsequently, a principal component analysis (PCA) with varimax rotation was performed using the remaining items. The suitability of the items for conducting a PCA was evaluated by applying the Kaiser‒Meyer‒Olkin (KMO) criterion. Various criteria, such as interpretability, scree plot, and explained total variance by the principal component exceeding 10%, were combined to identify the most relevant factors. To further reduce the items in the questionnaire, only items with factor loadings above 0.6 were retained.

To assess the internal consistency and reliability of the questionnaire using the remaining items, Cronbach’s alpha was calculated. Moreover, an overall score of the EFA23 questionnaire was calculated by summing up the values of the final selected items exhibited in values from 0, indicating “no limitations”, to 69, representing “complete limitations” in overall functioning.

To evaluate the construct validity of the EFA23 questionnaire, Pearson correlations (critical values: *r* > 0.5 high, *r* > 0.3 < 0.5 moderate, 0.3 < low [31]) were computed between the overall scale of the EFA23 questionnaire and the overall scales of the supplementary assessment tools (IADL, WHODAS, MOSES, STEP, and FAM). Further, we tested the correlation between the overall scale of the EFA23 questionnaire and the number of doctor visits using Spearman’s rank coefficient. Pearson’s coefficient was used to measure the correlation between the overall scale of the EFA23 and the level of care.

All statistical analyses were performed using IBM SPSS Statistics version 28.0.

### External GP assessment

To determine whether the respondents’ self-assessment corresponded with an assessment of their GPs, the latter were simultaneously asked to complete the EFA23. Although the EFA23 questionnaire was developed as a self-assessment tool for patients, we wanted to test the differences between the self- and external assessment. The Wilcoxon test was used to measure the differences.

### Development of a GP manual

Since the EFA23 questionnaire should support communication processes in the consultation, a GP manual was developed to interpret the questionnaire results and discuss them with the patients. Based on the findings from the consensus study [20], this manual incorporates contextual factors (personal and environmental factors) based on the ICF. Contextual factors were considered relevant by the consensus conference experts to address functioning but were too comprehensive to also be included in the questionnaire.

The primary objective of the manual is to enable GPs to extract insights and identify areas for intervention from the questionnaire. Thus, a 90-minute workshop involving a group of GPs was conducted in October 2022 to collaboratively create the manual. The manual encompasses the visualization of questionnaire results, as well as the provision of stimulating ideas and actionable recommendations.

## Results

### Item development

Out of the 51 selected ICF categories in the consensus process [20], we formulated 69 items for the draft of the EFA23 questionnaire.

### Qualitative pretest

In total, 15 participants (phase one: three one-on-one interviews, one focus group with four respondents; phase two: three one-on-one interviews, one focus group with five respondents) consented to participate in the pretest. The participants’ characteristics can be found in Table 1.

**Table 1:** Participants‘ characteristics (qualitative pretest)

	*N* = 15 (%)
**Sex**	
Female	9 (60.00)
Male	6 (40.00)
**Age**	
Under 45 years	9 (60.00)
45–69 years	4 (26.67)
Over 70 years	2 (13.33)
**Education degree**	
Secondary school diploma	1 (6.67)
Middle maturity	4 (26.67)
A-levels	6 (40.00)
University degree	4 (26.67)
**One or more chronic diseases**	
Yes	10 (75.00)
No	5 (5.00)

Among the 69 items presented to the respondents, 35 were adequately retained without modification. One item, asking about the ability to build or maintain an intimate relationship, was discarded since all respondents agreed that this question would be too personal to be included in a self-report questionnaire. Furthermore, 31 items were discarded since their meaning was already more adequately covered in at least one of the retained items. The wording of the remaining two items was revised by the research team to align with the propositions that emerged from the interviews and focus groups, leading to a 37-item questionnaire in total. For all items, the answer options were considered suitable by the respondents. All participants agreed that for the target group aged 75 and above, the questionnaire should contain a maximum of 20–25 items.

### Validation study

A total of 252 respondents completed the survey, while 15 questionnaires with missing informed consent were excluded from the analysis. The total sample contained 237 older adults aged 75 and above. Table 2 shows the sociodemographic and disease-related variables of the sample of the validation study.

**Table 2:** Sociodemographic and disease-related variables (validation study)

	*N* = 237 (%)	
**Sex**		
Female	132	(55.7)
Male	105	(44.3)
**Age** (years)^a^	81.7	(± 4.53)
**Education degree**		
No graduation	3	(1.3)
Secondary school diploma	42	(17.7)
Middle maturity	40	(16.9)
Completed apprenticeship	34	(14.3)
A-levels	10	(4.2)
University degree	52	(21.9)
Missing	56	(23.6)
**One or more chronic diseases**		
Yes	180	(75.9)
No	54	(22.8)
Missing	3	(1.3)
**Degree of care**		
No degree of care	178	(75.1)
1	14	(5.9)
2	21	(8.9)
3	13	(5.5)
4	5	(2.1)
5	1	(0.4)
Missing	5	(2.1)
**Frequency of GP visits in the last year**		
Never	5	(2.1)
1–2 times	52	(21.9)
3–5 times	87	(36.7)
More than 5 times	88	(37.1)
I don’t know	1	(0.4)
Missing	4	(1.7)

Notes: ^a^Displayed as the mean (standard deviation)

Based on the 37 items of the EFA23 draft questionnaire, the descriptive statistics of the newly generated items measuring the limitations in functioning can be found in Table 3.

**Table 3:** Descriptive statistics preliminary items

Item		Min.	Max.	0*	1*	2*	3*	Mean (± SD)
**I am able to…**								
1	… participate in community life.	0	3	189	5	27	6	0.34 (0.79)
2	… read and understand texts.**	0	3	203	1	25	2	0.25 (0.68)
3	… move outside of my home.	0	3	197	3	21	13	0.36 (0.86)
4	… change my body position, e.g. get up from a chair.**	0	3	219	0	14	2	0.14 (0.54)
5	… communicate with others.**	0	3	224	0	8	2	0.09 (0.45)
6	… move around (with or without aids).	0	3	210	1	17	5	0.21 (0.67)
7	… take care of my finances.	0	3	197	14	13	7	0.26 (0.70)
8	… write texts.	0	3	209	6	14	6	0.22 (0.67)
9	… solve everyday problems.	0	3	199	2	24	9	0.33 (0.81)
10	… carry an object from A to B.	0	3	204	1	22	7	0.28 (0.76)
11	… take care of my health.	0	3	199	4	21	10	0.32 (0.81)
12	… calculate.**	0	3	219	3	10	3	0.14 (0.53)
13	… push something away with my foot.	0	3	212	2	16	6	0.22 (0.68)
14	… spend my leisure time actively.	0	3	190	5	18	19	0.42 (0.95)
15	… grab a small object.**	0	3	210	0	19	2	0.19 (0.61)
16	… move my hands and arms.**	0	2	223	0	13	0	0.11 (0.46)
17	… contact service providers, such as a hairdresser or doctor.	0	3	214	6	3	10	0.18 (0.66)
18	… drink without help.**	0	2	234	0	2	0	0.02 (0.18)
19	… drive a vehicle.	0	3	170	28	2	23	0.45 (0.94)
20	… communicate.**	0	2	227	0	7	0	0.06 (0.34)
21	… listen to other people.**	0	2	223	0	10	0	0.09 (0.41)
22	… prepare my food.	0	3	200	11	13	7	0.25 (0.70)
23	… cope with stress.	0	3	183	8	28	8	0.39 (0.84)
24	… go up and down stairs.	0	3	190	5	20	18	0.42 (0.94)
25	… do my shopping.	0	3	187	12	12	17	0.38 (0.89)
26	… maintain relationships with family members/ acquaintances/friends.	0	3	217	1	14	2	0.15 (0.55)
27	… eat without help.**	0	2	231	0	3	0	0.03 (0.23)
28	… stay in one body position for a longer time.**	0	3	210	1	13	4	0.17 (0.60)
29	… put on clothes.	0	3	217	2	11	4	0.15 (0.57)
30	… use the toilet.**	0	3	225	0	5	3	0.08 (0.44)
31	… use a (mobile) phone.	0	3	206	11	11	4	0.19 (0.60)
32	… take care of my body.	0	3	210	3	16	3	0.19 (0.61)
33	… watch other people doing activities.**	0	2	230	0	2	0	0.02 (0.19)
34	… manage my household.	0	3	186	13	21	10	0.37 (0.82)
35	… move inside my home.**	0	3	219	0	12	3	0.14 (0.55)
36	… deal with crises.	0	3	183	6	30	8	0.40 (0.85)
37	… make new acquaintances/friends.	0	3	198	18	8	10	0.27 (0.73)

***Notes***: Frequencies partly do not result in 237 persons due to missing data

*Response frequency of Scale (0 = no limitation; 3 = high limitation)

**Items that were removed from the questionnaire in the process of validation

In the first step, we excluded eleven items, as the range of possible answers for those items was not fully used. Principal component analysis (PCA) with varimax rotation was calculated with the remaining items. The Kaiser‒Meyer‒Olkin criterion was 0.925, and the Bartlett test was highly significant (*p* < 0.001), so the items were suitable for conducting a PCA. The first component accounted for 53.15% of the variance, while the other five components explained less than 10% of the variance. Upon examining the (rotated) component matrix, clear and high loadings were observed only for the first component. Additionally, the scree plot revealed a bend at the second component. This indicates the presence of one significant principal component, named “Limitations in functioning”.

Considering a factor loading cutoff point of 0.6 to further reduce the questionnaire, we removed three additional items with a factor loading < 0.6. The factor loadings of the remaining items can be found in additional file 1. After performing PCA again with a fixed factor and the reduced number of items, the explained total variance increased to 56.55%. The internal consistency of the final EFA23 questionnaire, consisting of 23 items, yielded a Cronbach’s alpha of 0.967. Testing the construct validity showed positive correlations between the total score of the EFA23 questionnaire and the respective total scores of the selected assessment tools (Table 4, additional file 2).

**Table 4:** Pearson correlations between the EFA23 overall scale and other assessment tools

	EFA23
IADL	0.772
WHODAS	0.856
MOSES	0.783
STEP	0.498
FAM	0.701

We found a statistically significant Spearman’s *ρ* = 0.464 (*p* < 0.001) between the overall score of the EFA23 questionnaire and the number of visits to the doctor. The overall score of the EFA23 and the level of care showed a significant moderate correlation (*r* = 0.591, *p* < 0.001) [31].

### External GP assessment

Of the 38 participants who were recruited via their GP, 26 gave their consent to an external assessment by their GP. An ANOVA for independent samples showed that respondents with and without external evaluation did not differ significantly from each other in terms of their limitation overall score of the EFA23 questionnaire, *F*(1, 179) = 0.073, *p* = 0.788. We tested the two limitation overall scores of the self-assessment and the external GP assessment for normal distribution. Both scores were not normally distributed according to the Shapiro‒Wilk test (*p* < 0.001). Therefore, we applied the Wilcoxon test, a nonparametric method, to test whether the two overall scores differed statistically significantly. Descriptive statistics showed differences between the means of the self-assessment = 7.19 (*SD* = 1.2) compared to the external GP assessment = 0.33 (*SD* = 11.9). However, this difference was not statistically significant (*z* = -0.677, *p* = 0.498).

### Development of a GP manual

Five GPs participated in the workshop to develop the manual. As in the preparatory study from the experts’ perspective [18], the workshop respondents identified environmental factors from various categories of the ICF. Consequently, it was decided to include exemplary questions on environmental factors that can support GPs in discussing the EFA23 questionnaire with their patients, supplemented by personal factors to cover the contextual factors of the ICF in the manual. The manual advice on how to deal with the EFA23 questionnaire. If patients indicate more than three limitations, GPs should ask which two to three problems are currently the most relevant or important to focus the consultation on first.

## Discussion

To our knowledge, this is the first time a self-assessment tool for community-dwelling older adults to focus on functioning in general practice has been developed entirely based on the ICF following the methodological approach proposed by the ICF Research Branch. There are notable differences between the EFA23 questionnaire and other assessment tools measuring functional health. The STEP Assessment and its shorter version, the *Manageable Geriatric Assessment* (MAGIC), include questions and tests for common health problems of older people living independently [32–34]. However, both assessment tools have been developed solely from the perspective of experts or researchers. Although the *Oxford Participation and Activities Questionnaire* (Ox-PAQ) and MOSES questionnaire are based on the theoretical framework of the ICF, the selection of relevant categories did not follow the recommended methodology of the ICF Research Branch and were not developed specifically for older patients [27, 35].

The ICF provides a suitable framework for capturing functioning; nevertheless, in this study, we made the decision not to encompass all of its components. During the consensus study discussions, it was determined that body functions and structures were already comprehensively assessed in general practice. However, there may be gaps in the assessment of contextual factors, activities, and participation, which are crucial for obtaining a comprehensive understanding of patients’ functioning [20]. Therefore, the EFA23 questionnaire predominantly focuses on activities and participation, while the GP manual supplements this by including contextual factors.

A strength of this study is the assessment of the psychometric properties of the EFA23 questionnaire, which measures activities and participation by addressing relevant aspects for the target group. The development process incorporated different perspectives, including those of research, patients, experts, and clinicians, with patients being involved in multiple stages (preparatory phase, qualitative pretest, and validation study) to ensure suitability for the target group. The qualitative pretest permitted improvement in terms of the clarity of the items. The validation study showed that the ICF-based items are suitable for assessing functioning in a patient self-report questionnaire. Finding one principal component as a result of the PCA underlines the need for a holistic perspective when addressing functioning.

There are also some limitations to be considered. In the pretest, we decided not to conduct interviews exclusively with older patients, which may have led to a possible bias. Nevertheless, we assume that since the patients’ perspective in the preparatory phase and in the validation study included only people aged 75 and older, their perspective was sufficiently covered. The validation phase may have also suffered from a slight selection bias since we are unaware of the motivations of the respondents to participate in the validation study. Moreover, since the survey in the validation study also included sociodemographic and disease-related questions in addition to the EFA23 questionnaire, along with supplementary assessment tools, it might have been a burden for some patients to participate in the study in terms of a larger number of questions to be answered. We found a difference between the mean values of the patients’ self-assessment and the GPs’ external assessment, whereby the GPs seem to underestimate the limitations in functioning. However, this difference was not statistically significant, which may be due to the small sample size of the external assessments, and the questionnaire was not developed as an external assessment tool.

## Conclusions

The validation of the German EFA23 questionnaire has led to a self-assessment tool aiming to open up and support conversations about functional health in the consultation. Its development followed a methodological approach by the ICF research branch, involving multiple perspectives, ensuring suitability for the target group. The aim of using the EFA23 questionnaire in general practice is to address the unique challenges posed by multimorbidity in older adults and shift to a more person- and context-centred approach, considering not only disease-related information but also information about functioning in terms of activities and participation.

### Electronic supplementary material

Below is the link to the electronic supplementary material.

Supplementary Material 1

Supplementary Material 2

Supplementary Material 3

Supplementary Material 4

Supplementary Material 5

Supplementary Material 6

## Data Availability

The German EFA23 questionnaire and GP manual are present in additional information files. An English translation of the questionnaire (not validated) and GP manual are also present in the supplementary information files. The full datasets used and analysed during the validation study are available from the corresponding author on reasonable request.

## Abbreviations

ICF: International Classification of Functioning, Disability and Health
EFA23: Erfassung Funktionaler Gesundheit im Alter – 23 Fragen/Assessing Functional Health in Old Age – 23 questions
GP: General Practitioner
IADL: Instrumental activities of daily living scale
WHODAS 2.0: World Health Organization Disability Assessment Schedule
MOSES: Questionnaire to assess the areas Mobility, Self-Care and Domestic life
STEP: Standardized assessment of elderly people in primary care
FAM: Functional Assessment Measure
PCA: Principal Component Analysis
KMO: Kaiser‒Meyer‒Olkin
MAGIC: Manageable Geriatric Assessment
Ox-PAQ: Oxford Participation and Activities Questionnaire
