# Supplementary material for: Development of a self-assessment tool to address the functioning of community-dwelling older adults in general practice: a validation study of the EFA23 questionnaire
Source: BMC Prim Care. 2024 Aug 2;25:280. doi: 10.1186/s12875-024-02539-6 (PMC11297772; doi:10.1186/s12875-024-02539-6)
Supplement: Supplementary file 1 — Supplementary Material 1 [file 12875_2024_2539_MOESM1_ESM.pdf]

FACTOR LOADINGS	
I am able to …	Principal component 'Limitations in functioning'
… spend my leisure time actively.	0.864
… prepare my food.	0.848
… do my shopping.	0.845
… manage my household.	0.816
… take care of my health.	0.804
… move outside of my home.	0.802
… go up and down stairs.	0.800
… solve everyday problems.	0.783
… contact service providers, such as a hairdresser or doctor.	0.777
… make new acquaintances/friends.	0.771
… take care of my body.	0.765
… move around (with or without aids).	0.756
… put on clothes.	0.749
… maintain relationships with family members/acquaintances/friends.	0.749
… carry an object from A to B.	0.733
… take care of my finances.	0.714
… push something away with my foot.	0.702
… write texts.	0.689
… deal with crises.	0.672
… drive a vehicle.	0.667
… participate in community life.	0.665
… cope with stress.	0.649
… use a (mobile) phone.	0.601

Principal component analysis.
