# Supplementary figures and images for: Development of a self-assessment tool to address the functioning of community-dwelling older adults in general practice: a validation study of the EFA23 questionnaire
Source: BMC Prim Care. 2024 Aug 2;25:280. doi: 10.1186/s12875-024-02539-6 (PMC11297772; doi:10.1186/s12875-024-02539-6)

Correlation plot between EFA23 overall scale and other assessment tools

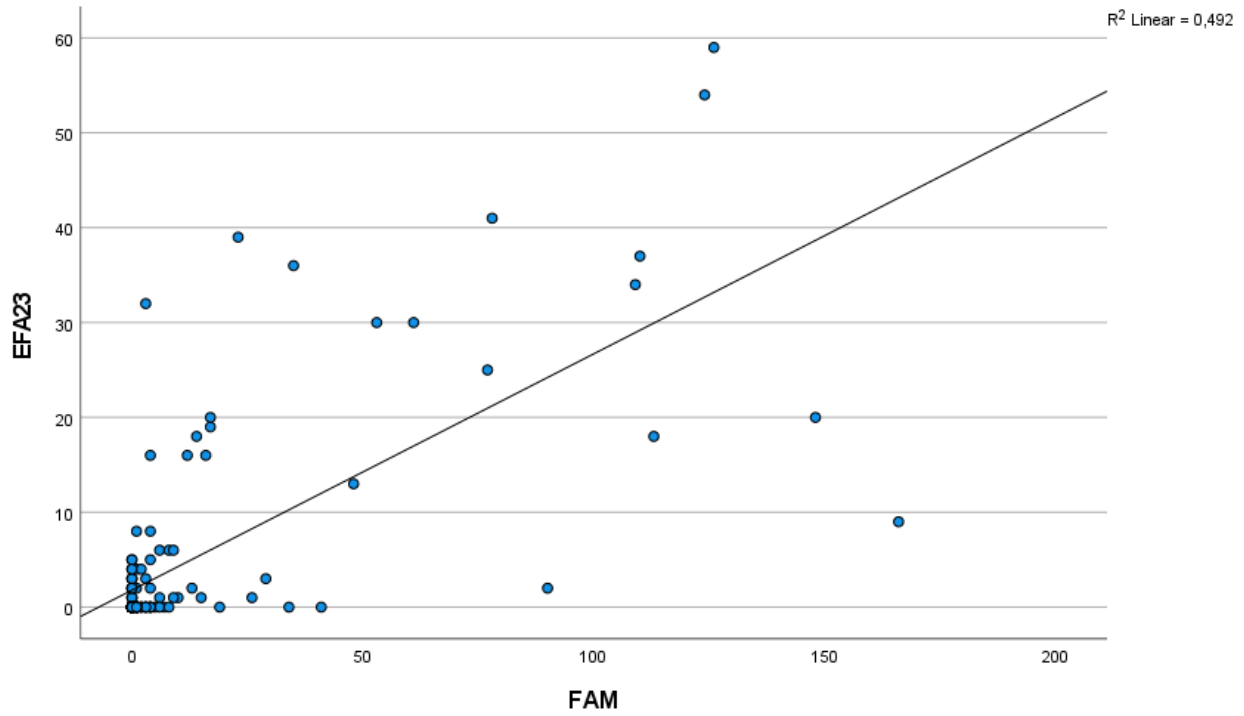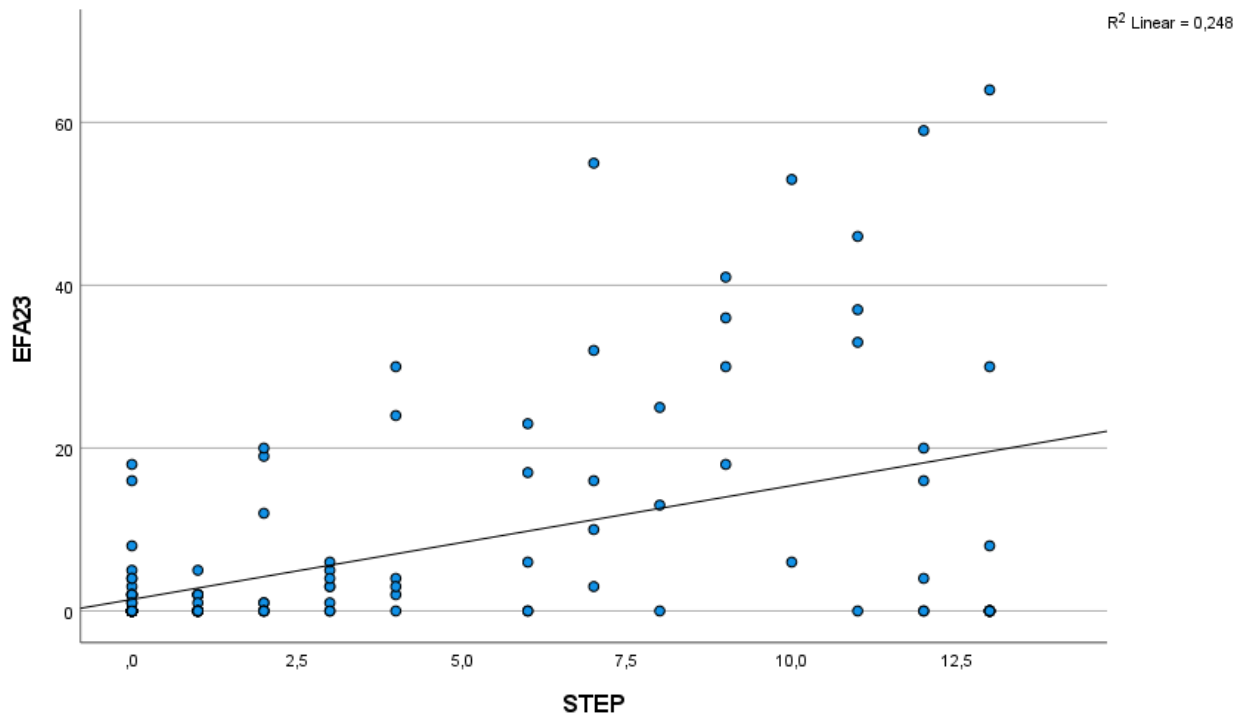

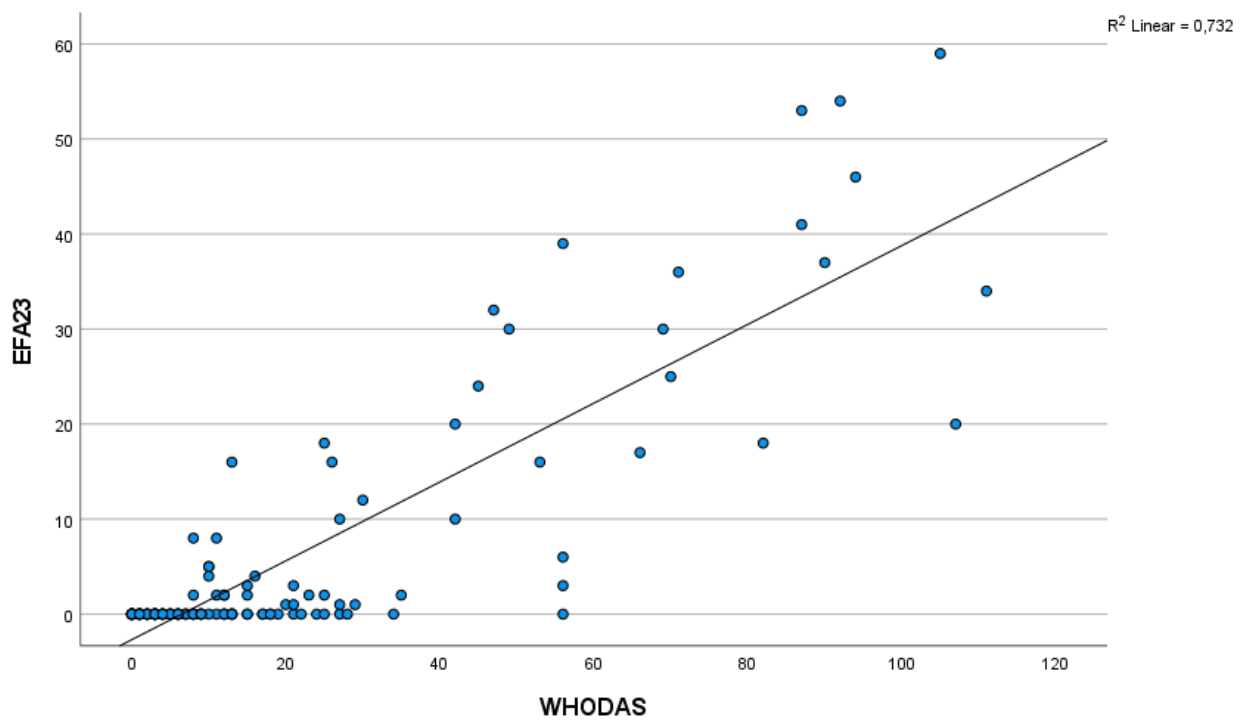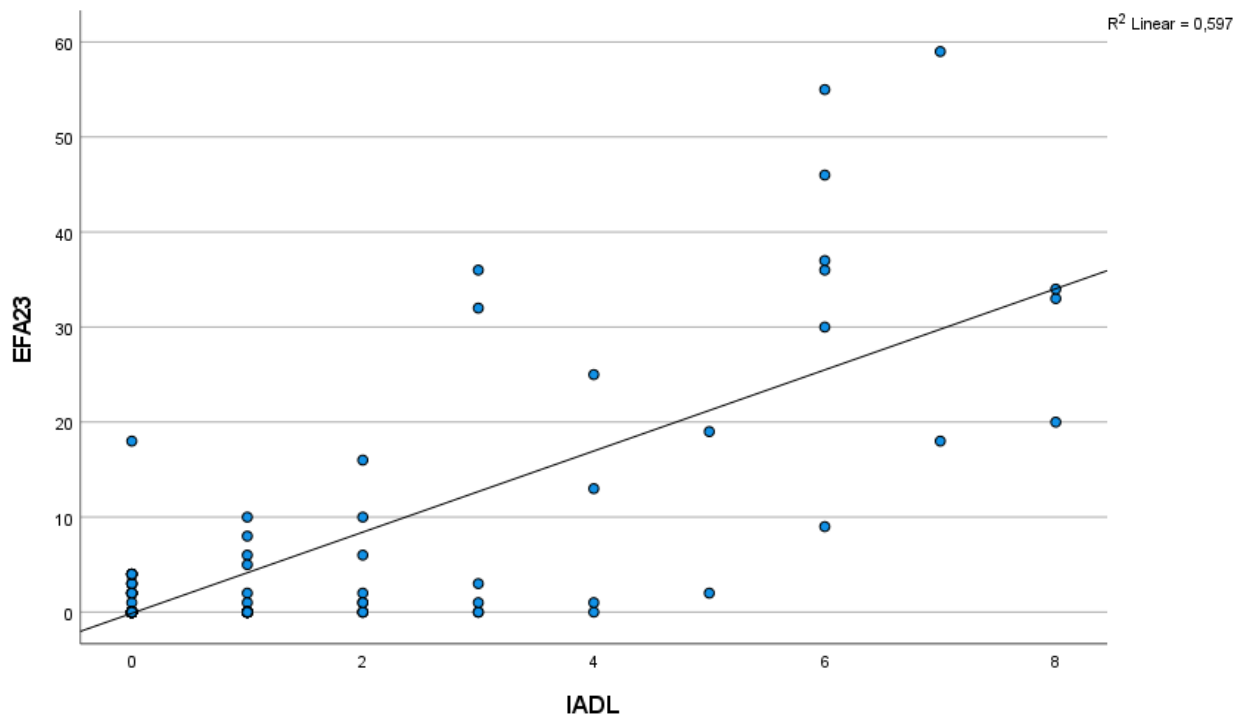

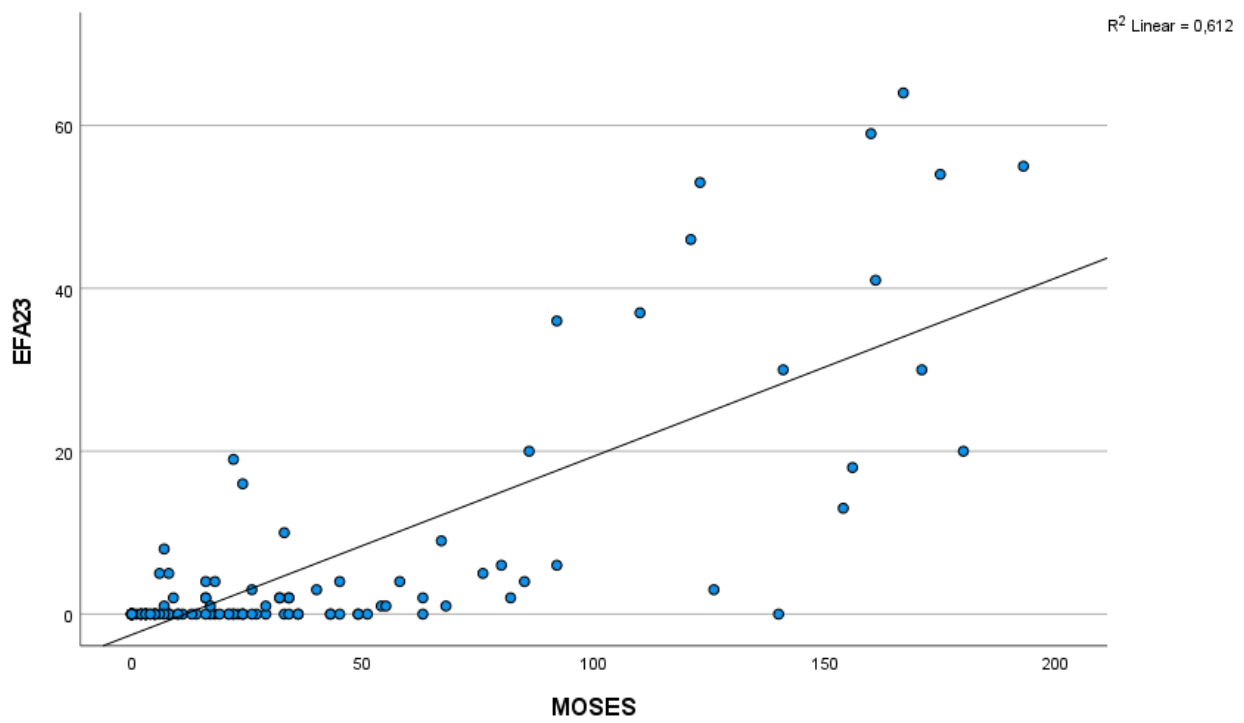

Supplement: Supplementary file 2 — Supplementary Material 2 [file 12875_2024_2539_MOESM2_ESM.pdf]
