# Supplementary material for: Development of a self-assessment tool to address the functioning of community-dwelling older adults in general practice: a validation study of the EFA23 questionnaire
Source: BMC Prim Care. 2024 Aug 2;25:280. doi: 10.1186/s12875-024-02539-6 (PMC11297772; doi:10.1186/s12875-024-02539-6)
Supplement: Supplementary file 3 — Supplementary Material 3 [file 12875_2024_2539_MOESM3_ESM.pdf]

EFA23 (Erfassung Funktionaler Gesundheit im Alter – 23 Fragen)

Fragebogen für Personen ab 75 Jahren in der hausärztlichen Praxis

Bitte geben Sie an, welche Aktivitäten Sie durchführen **können.** Es geht nicht darum, ob Sie die Aktivität gerne oder häufig durchführen, sondern nur, ob Sie diese ausüben könnten.
Bewerten Sie anschließend, ob die Einschränkungen für Sie im Alltag ein Problem darstellen.

**Ich kann…**		**Ja**	**Teil-weise**	**Nein**	Bei Teilweise/Nein: **Ist das ein Problem für Sie?**	
					**Ja**	**Nein**
1.	… Texte schreiben.					
2.	… alltägliche Probleme lösen.					
3.	… mit Stress umgehen.					
4.	… mit Krisen umgehen.					
5.	… ein (Mobil-)Telefon benutzen.					
6.	… einen Gegenstand von A nach B tragen.					
7.	… etwas mit dem Fuß wegschieben.					
8.	… mich (mit oder ohne Hilfsmittel) fortbewegen.					
9.	… Treppen auf- und absteigen.					
10.	… mich außerhalb meines Zuhauses fortbewegen.					
11.	… ein Fahrzeug fahren.					
12.	… meinen Körper pflegen.					
13.	… mich anziehen.					
14.	… mich um meine Gesundheit kümmern.					
15.	… meine Einkäufe erledigen.					
16.	… mein Essen zubereiten.					
17.	… meinen Haushalt führen.					
18.	… Kontakt zu Dienstleistern, z.B. einem Friseur oder Arzt, aufnehmen.					
19.	… neue Bekanntschaften machen/Freundschaften schließen.					
20.	… Beziehungen zu Familienmitgliedern/Bekannten/Freunden pflegen.					
21.	… mich um meine Finanzen kümmern.					
22.	… am gemeinschaftlichen Leben teilnehmen.					
23.	… meine Freizeit aktiv gestalten.					
