# Supplementary material for: Development of a self-assessment tool to address the functioning of community-dwelling older adults in general practice: a validation study of the EFA23 questionnaire
Source: BMC Prim Care. 2024 Aug 2;25:280. doi: 10.1186/s12875-024-02539-6 (PMC11297772; doi:10.1186/s12875-024-02539-6)
Supplement: Supplementary file 4 — Supplementary Material 4 [file 12875_2024_2539_MOESM4_ESM.pdf]

EFA23 (Erfassung Funktionaler Gesundheit im Alter)
Manual für Hausärzt*innen ergänzend zum EFA23 Fragebogen

Mit zunehmenden Alter steigt zumeist auch die Anzahl der Erkrankungen. Ein rein krankheitsorientierter Ansatz stößt hier meist an seine Grenzen und bringt u.a. Polypharmazie mit sich.

Ein stärker personen- und kontextbezogener Ansatz ist erforderlich, der sich auf die funktionale Gesundheit und auf die Folgen von Multimorbidität auf das alltägliche Leben konzentriert.

Ziel des EFA23 Fragebogens ist die Erfassung von Problembereichen funktionaler Gesundheit von Patient*innen ab 75 Jahren. Der Fragebogen soll Sie unterstützen, Probleme mit Ihren Patient*innen zu besprechen und gemeinsam alltagsrelevante Therapieziele festzulegen.

Lassen Sie den Fragebogen von den Patient*innen ab 75 Jahren vorab im Wartezimmer ausfüllen.
Nutzen Sie die Antworten und angegebenen Problembereiche als Gesprächseinstieg.
Unterstützend zum Fragebogen finden Sie im Folgen weitere Bereiche und Hinweise, die die Besprechung unterstützen können.

i Wenn mehr als 3 Problembereiche im Fragebogen angegeben wurden, bitten Sie den/die Patient*in die 2-3 aktuell relevantesten/wichtigsten Alltagsprobleme zu benennen, um diese zu besprechen.

Zur Besprechung des EFA23 Fragebogens können folgende Einstiegsfragen dienen:
Gibt es Dinge, die Sie im Alltag behindern?
Oder Dinge, die Ihre Probleme weniger werden lassen?

Versorgung

Wie kommen Sie mit Ihren Medikamenten klar?

Gibt es Hilfsmittel, die Ihren Alltag erleichtern würden?

Lebenswelt

Wie ist ihre aktuelle Wohnsituation?

Wie ist Ihre infrastrukturelle Anbindung?

Bereiten Ihnen bestimmte Temperaturen/ Wetterlagen Schwierigkeiten?

Unterstützung

Wie ist Ihre Einstellung zu Familie und Freunden?

Gibt es Personen, die Sie im Alltag unterstützen?

Würden Sie sich mehr Unterstützung wünschen? Von wem?

Umstehend finden Sie mögliche Fragen zu **persönlichen Faktoren** und **Umweltfaktoren**, die das Gespräch unterstützen können.

Alle Bereiche können einen lösenden, aber auch einen behindernden Einfluss auf die Problembereiche haben.

Persönliche Faktoren

Berücksichtigt werden sollten auch Faktoren wie: Lebensstil, sozialer Hintergrund und Charakterzüge der Patient*innen.
