# Supplementary material for: Development of a self-assessment tool to address the functioning of community-dwelling older adults in general practice: a validation study of the EFA23 questionnaire
Source: BMC Prim Care. 2024 Aug 2;25:280. doi: 10.1186/s12875-024-02539-6 (PMC11297772; doi:10.1186/s12875-024-02539-6)
Supplement: Supplementary file 5 — Supplementary Material 5 [file 12875_2024_2539_MOESM5_ESM.pdf]

EFA23 (Assessing Functional Health in Old Age – 23 questions)

Questionnaire for people aged 75 and above in general practice

Please indicate which activities you **are able to do**. It is not a matter of whether you like to do the activity or do it often, but only whether you could do it.<br>Then rate whether the limitations are a problem for you in everyday life.						
**I am able to …**		**Yes**	**Partly**	**No**	If Partly/No: **Is this a problem?**	
					**Yes**	**No**
1.	… write texts.					
2.	… solve everyday problems.					
3.	… cope with stress.					
4.	… deal with crises.					
5.	… use a (mobile) phone.					
6.	… carry an object from A to B.					
7.	… push something away with my foot.					
8.	… move around (with or without aids).					
9.	… go up and down stairs.					
10.	… move outside of my home.					
11.	… drive a vehicle.					
12.	… take care of my body.					
13.	… put on clothes.					
14.	… take care of my health.					
15.	… do my shopping.					
16.	… prepare my food.					
17.	… manage my household.					
18.	… contact service providers, such as a hairdresser or doctor.					
19.	… make new acquaintances/friends.					
20.	… maintain relationships with family members/acquaintances/friends.					
21.	… take care of my finances.					
22.	… participate in community life.					
23.	… spend my leisure time actively.					
