# Supplementary material for: Development of a self-assessment tool to address the functioning of community-dwelling older adults in general practice: a validation study of the EFA23 questionnaire
Source: BMC Prim Care. 2024 Aug 2;25:280. doi: 10.1186/s12875-024-02539-6 (PMC11297772; doi:10.1186/s12875-024-02539-6)
Supplement: Supplementary file 6 — Supplementary Material 6 [file 12875_2024_2539_MOESM6_ESM.pdf]

EFA23 (Assessing Functional Health in Old Age – 23 questions)

Manual for GPs complementary to the EFA23 questionnaire

With increasing age, the number of diseases often increases accordingly. A purely disease-oriented approach often reaches its limits and can lead to polypharmacy, among other issues.

A more person-centered and context-specific approach is necessary, focusing on functional health and the consequences of multimorbidity on daily life.

The aim of the EFA23 questionnaire is to assess problem areas related to functional health in patients aged 75 and above. The questionnaire is designed to assist you in discussing issues with your patients and collaboratively setting everyday-relevant therapy goals.

Ask patients aged 75 and over to complete the questionnaire in the waiting room.
Use the answers and problem areas identified as a starting point for the consultation.
In addition to the questionnaire, you will find further areas and advice in the following that may support the consultation.

i If more than 3 problem areas are indicated in the questionnaire, ask the patient to identify the current 2-3 most relevant/important everyday problems to have a consultation about.

The following initial questions can be helpful to discuss the EFA23 questionnaire:
Are there things that hinder you in your everyday life?
Or things that make your problems less?

Supply

How do you cope with your medication?

Are there any aids that would make your everyday life easier?

Living environment

What is your current housing situation?

What are your infrastructural accessibilities?

Do certain temperatures/ weather conditions cause you difficulties?

Support

What is your attitude to family and friends?

Are there people who support you in everyday life?

Would you like more support? From whom?

Around you will find potential questions about **personal factors** and **environmental factors** that can support the conversation.

All areas can have a solving, but also a hindering influence on the problem areas.

Personal factors

Consideration should also be given to factors such as: lifestyle, social background and character attributes of the patients.
